# Complex MIMO RBF Neural Networks for Transmitter Beamforming over Nonlinear Channels

**DOI:** 10.3390/s20020378

**Published:** 2020-01-09

**Authors:** Kayol Soares Mayer, Jonathan Aguiar Soares, Dalton Soares Arantes

**Affiliations:** Department of Communications, School of Electrical and Computer Engineering, University of Campinas, Campinas 13083-852, Brazil; jonathan@decom.fee.unicamp.br (J.A.S.); dalton@decom.fee.unicamp.br (D.S.A.)

**Keywords:** artificial neural networks, radial basis function networks, complex-valued kernel, nonlinear transmitter beamforming, adaptive array.

## Abstract

The use of beamforming for efficient transmission has already been successfully implemented in practical systems and is absolutely necessary to even further increase spectral and energy efficiencies in some configurations of the next-generation wireless systems and for low earth orbit satellites. A remarkable capacity increase is then achieved and spectral congestion is minimized. In this context, this article proposes a novel complex multiple-input multiple-output radial basis function neural network (CMM-RBF) for transmitter beamforming, based on the phase transmittance radial basis function neural network (PTRBFNN). The proposed CMM-RBF is compared with the least mean square (LMS) algorithm for beamforming with six dipoles arranged in a uniform and circular array and with 16 dipoles in a 2D-grid array. Simulation results show that the proposed solution presents lower steady-state mean squared error, faster convergence rate and enhanced half-power beamwidth (HPBW) when compared with the LMS algorithm in a nonlinear scenario.

## 1. Introduction

In the last decades, artificial neural networks (ANNs) have attracted much attention, performing specific tasks in different applications, such as clustering, prediction, classification, pattern recognition, machine learning and artificial intelligence. As ANNs are mainly designed to mimic the human brain, a considerable number of approaches only handle real-valued signals [1,2,3,4]. However, some engineering problems are intrinsically dependent on complex-valued signals (e.g., channel equalization and beamforming). In order to circumvent this limitation, ANN algorithms based on complex numbers have already been proposed for some applications, such as channel equalization [5,6,7] and adaptive beamforming for wireless receivers [8,9,10,11].

Digital communication systems over wireless channels may suffer severe signal distortions due to multipath propagation, additive white Gaussian noise (AWGN) [12,13], Doppler effects and, not infrequently, nonlinearities at the receiver front-end and at the transmitter high power amplifier [5,7]. Since nonlinear impairments usually worsen the performance of linear channel equalizers, nonlinearities in the channel are better dealt with using robust nonlinear equalizers [5,7,14]. In this context, based on the phase transmittance radial basis function neural network (PTRBFNN) equalizer [7], a blind fuzzy controller algorithm was applied to increase the concurrent neural network equalizer (CNNE) convergence speed and decrease the residual mean squared error (MSE) [5]. Another equally important technique is the butterfly neural equalizer (BNE) which, when applied to optical communications with two-dimensional digital modulation, is able to mitigate nonlinearities in the photo-electric converters and simultaneously compensate chromatic and polarization mode dispersions [6].

For beamforming at the receiver, ANN architectures such as the bi-dimensional neural beamformer with joint error (BNB-JE), the butterfly neural beamformer (NB-Butterfly), and the beamformer neural network (BNN) are potential algorithms for improving receiver performance [9,10,11]. On the other hand, beamforming for efficient transmission is necessary to increase spectral and energy efficiencies in some configurations of the next-generation wireless systems [15]. In addition, for low earth orbit (LEO) satellites, the use of beamforming techniques is economically important to mainly reduce the power consumption and to increase the data throughput [16,17]. Also, nonlinear beamfoming algorithms can play a key role for band-limited systems employing nonlinear power amplifiers, as in satellite communication systems [18].

In communication systems with beamforming at the transmitter, arrays with several antennas are applied to focus the electromagnetic signal towards the desired receiver [19]. These arrays can be controlled by three different architectures: digital, analog, and hybrid. Usually, in digital beamforming the channel knowledge is required at the transmitter and some precoding technique is necessary [20,21,22,23,24,25], impacting the hardware with very high computational complexity and energy consumption. [26]. On the other hand, in analog beamforming the RF signals are manipulated by means of controlling phase shifters and/or variable gain amplifiers (VGAs). Although this architecture has low computational complexity and power consumption, it is less flexible and presents inferior results when compared with digital beamforming [26,27].

In the context of LEO satellites, the use of the classical digital beamforming techniques [20,21,22,23,24,25] is prohibitive due to the power and computational complexity constraints, which is why analog beamforming techniques are employed in this area [16,17]. However, by means of a digital beamforming without channel knowledge and precoding, as proposed here, it is possible to perform a low power digital architecture which is more flexible than the analog one. This problem can be modeled as a set of electric currents whose phases and amplitudes are modulated in such a way that the antenna radiation pattern points to the correct direction. A useful method to determine the beamformer electric currents is via the least mean square (LMS) algorithm. In this linear method, the filter weights, which represent the array of electric currents, are updated by a convex cost function [28] to minimize the error between the obtained and the desired radiation patterns [29]. However, LEO satellites frequently operate with high power amplifiers which suffer severe signal distortion due to the nonlinearities [18]. This nonlinear scenario reduces the LMS performance because of its linear design.

Differently from the LMS, neural networks can operate like nonlinear filters [30]. The nonlinear structure of a neural network is modeled by nonlinear activation functions in multilayer perceptrons (MLPs) or by Gaussian neurons in radial basis function neural networks (RBFNNs) [30]. The RBFNNs Gaussian neurons have two free parameters, namely the Gaussian centers and the variances. Besides, there is a linear free vector parameter of weights, which linearly weighs the output of the neurons to yield the network output [7]. Via these three free parameters, RBFNNs can represent high-order nonlinear spaces without the necessity of increasing the number of layers, reducing its complexity in comparison with deep neural networks. Although artificial neural networks have been employed for beamforming, the ANN architectures presented in the literature are unfeasible for the proposed application, since they are designed for beamforming in receiver devices or require channel information and/or precoding.

In such context, this article proposes a novel architecture of RBFNN, based on a complex multiple-input multiple-output (MIMO) RBFNN (CMM-RBF) for beamforming transmitters, in contrast to [9,10,11] which are designed to beamforming receivers. The proposed system applies a MIMO variation of the multiple-input single-output (MISO) phase transmittance RBFNN (PTRBFNN) [7] to a beamforming structure to generate a unified nonlinear solution. The PTRBFNN model was chosen due its lower computational complexity in comparison with deep neural networks, and due to its important role in avoiding any phase invariance at the output of the neurons in comparison with a complex RBFNN [7]. Results show that the proposed architecture achieves enhanced half-power beamwidth (HPBW), faster convergence rate and lower steady-state mean squared error (MSE) when compared with LMS beamforming in a nonlinear scenario.

The remainder of this article is organized as follows. In Section 2, a mathematical modeling is described for a general arrangement of antennas. The LMS algorithm and the proposed complex MIMO radial basis function for beamforming are presented in Section 3 and Section 4, respectively. In Section 5, simulation results of the CMM-RBF are compared to results obtained by LMS, considering half-power beamwidth (HPBW), steady-state mean squared error, and convergence rate in a nonlinear scenario. Conclusions are discussed in Section 6.

## 2. Antenna Array Modeling

In a transmitter, when operating with an antenna array with *P* dipoles of length *l* and an arrangement of *Q* sensors around the antenna array, the matrix of steering vectors $\Psi ={\left[\psi_{1}^{T}\;\psi_{2}^{T}\;\cdots \;\psi_{Q}^{T}\right]}^{T}$ is

$$\Psi =\zeta V\;\in \;C^{Q\times P},$$

in which ${\left[\cdot \right]}^{T}$ is the transpose operator. The *q*th steering vector $\psi_{q}\in C^{P\times 1}$ expresses the radiation pattern towards the *q*th sensor. The matrix of relative intensity of the electric field $\zeta \in R^{Q\times Q}$ is

(1) $$\zeta =\left[\begin{array}{cccc} \zeta_{1} & 0 & \cdots & 0\\ 0 & \zeta_{2} & \cdots & 0\\ \vdots & \vdots & \ddots & \vdots \\ 0 & 0 & \cdots & \zeta_{Q} \end{array}\right].$$

**Figure 1:**
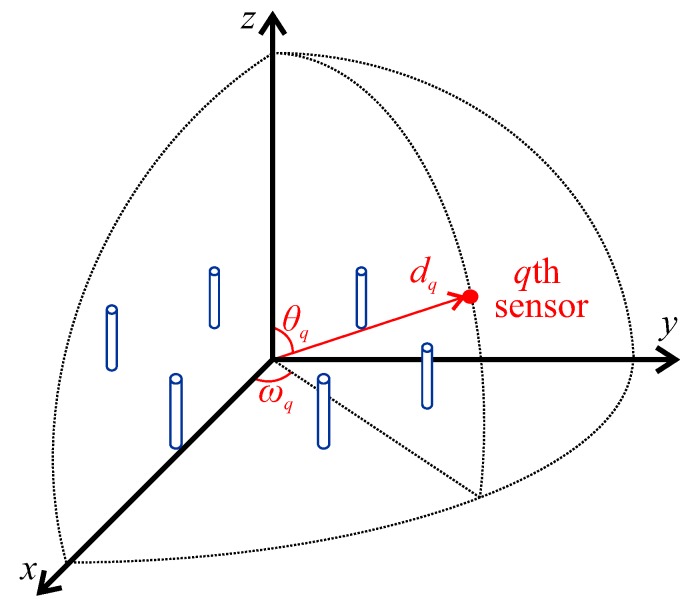
Representation of the angular position of the *q*th sensor around the array of antennas.

The *q*th element of the main diagonal of ζ is

$$\zeta_{q}=\frac{\cos\left(\pi l\cos\left(\theta_{q}\right)\lambda^{-1}\right)-\cos\left(\pi l\lambda^{-1}\right)}{\sin(\theta_{q})},$$

in which λ=c/f is the signal wavelength, c=299,792,458 m/s is the speed of light in vacuum, *f* is the frequency of the transmitted signal, and $\theta_{q}$ is the zenith angle of the *q*th sensor. Figure 1 presents the angular position of the *q*th sensor ($\theta_{q}$, $\omega_{q}$) and the related radiation pattern ($d_{q}$), for any arrangement of dipoles.

The matrix $V={\left[v_{1}^{T}\;v_{2}^{T}\;\cdots \;v_{Q}^{T}\right]}^{T}\in C^{Q\times P}$ is

$$V=\exp(𝚥2\pi \lambda^{-1}\Omega C),$$

where exp(·) is the scalar exponential function, $C\in R^{3\times P}$ is the matrix of Cartesian coordinates (*x*, *y*, *z*) of the dipoles:

(2) $$C=\left[\begin{array}{cccc} x_{1} & x_{2} & \cdots & x_{P}\\ y_{1} & y_{2} & \cdots & y_{P}\\ z_{1} & z_{2} & \cdots & z_{P} \end{array}\right],$$

and $\Omega \in R^{Q\times 3}$ is the sensors matrix of angular position:

(3) $$\Omega =\left[\begin{array}{ccc} \sin\theta_{1}\cos\omega_{1} & \sin\theta_{1}\sin\omega_{1} & \cos\theta_{1}\\ \sin\theta_{2}\cos\omega_{2} & \sin\theta_{2}\sin\omega_{2} & \cos\theta_{2}\\ \vdots & \vdots & \vdots \\ \sin\theta_{Q}\cos\omega_{Q} & \sin\theta_{Q}\sin\omega_{Q} & \cos\theta_{Q} \end{array}\right],$$

in which $\omega_{r}$ is the azimuth angle of the $r^{th}$ sensor.

Note that this modeling is applicable for any array setup in three dimensions, taking into account the matrix of Cartesian coordinates Equation (2) and the sensors matrix of angular position Equation (3). This Section was based on [31] (Chapter VI), in which the array equations are presented in a generalized matrix structure.

## 3. Least Mean Square Algorithm for Beamforming

**Figure 2:**
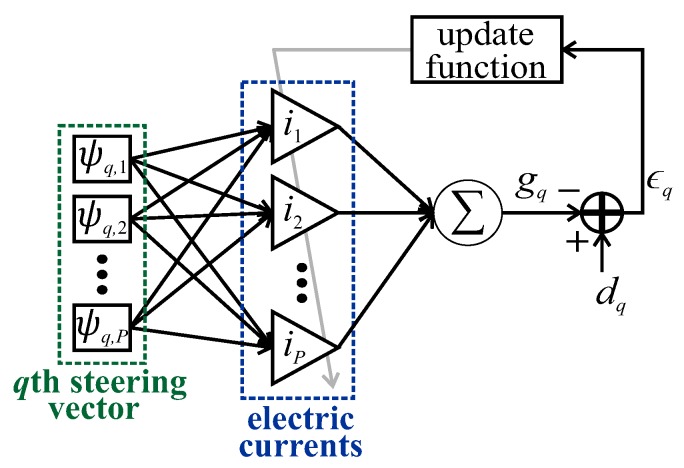
Least mean square (LMS) architecture for beamforming.

Considering a beamforming transmission with *P* antennas and an arrangement of *Q* sensors around the antenna array, then the vector of radiation pattern g towards the sensors is given by

$$g=\Psi i\;\in \;C^{Q\times 1},$$

where $i\in C^{P\times 1}$ is the vector of antenna electric currents. Figure 2 presents the LMS architecture for beamforming.

In order to control the array boresight, it is chosen a set of radiation conditions $d\in C^{Q\times 1}$, verified by *Q* sensors, which well describes the desired radiation pattern. Thus, with d, g and ψ, the LMS algorithm can be used to estimate i to the *q*th sensor at the *u*th training epoch by the minimization of the following cost function:

(4) $$J_{q}\left[u\right]=\frac{1}{2}{\left|d_{q}-g_{q}\left[u\right]\right|}^{2},$$

where |·| stands for absolute value and $\left(d_{q},g_{q}\right)$ are the *q*th target components of (d,g).

Thus, by means of the steepest descent algorithm, the update of the *p*th electric current of the LMS algorithm, to the *q*th steering vector, is given by:

(5) $$i_{p}\left[u\right]=i_{p}\left[u\right]-\eta_{l}\nabla^{i}J_{q}\left[u\right],$$

in which $\eta_{l}$ is the LMS adaptive step and $\nabla^{i}$ is the complex gradient operator of $i_{p}$.

Applying the complex gradient operators ($\nabla^{i}$) to Equation (4) yields:

(6) $$i_{p}\left[u\right]=i_{p}\left[u\right]+\eta_{l}\epsilon_{q}\left[u\right]\psi_{q,p}^{*},$$

where ${\left[\cdot \right]}^{*}$ denotes the complex conjugate operator and $\epsilon_{q}\left[u\right]=d_{q}-g_{q}\left[u\right]$ is the instantaneous error for the *q*th sensor at the *u*th training epoch. Finally, generalizing Equation (6) for i[u]:

$$i\left[u\right]=i\left[u\right]+\eta_{l}\epsilon_{q}\left[u\right]\psi_{q}^{*}.$$

In this LMS algorithm, each training epoch is composed of *Q* updates over i, and at the beginning of each training epoch i[u]≜i[u−1]. However, if u=0, then the vector of currents starts with i[0]≜0+𝚥0.

## 4. Complex MIMO Radial Basis Function Neural Network for Beamforming

**Figure 3:**
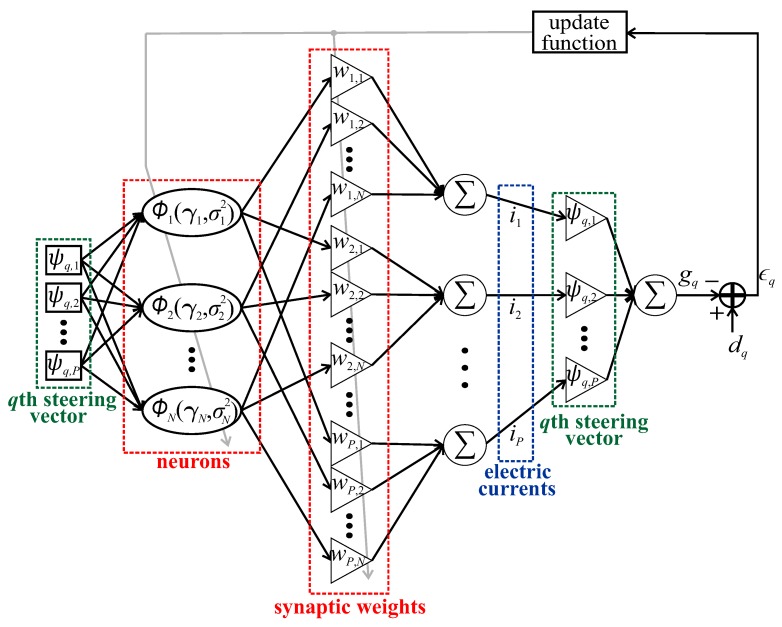
Complex multiple-input multiple-output radial basis function neural network architecture for beamforming.

As in the LMS beamforming technique, the input signal to the CMM-RBF algorithm is the set of steering vectors of Ψ, as shown in Figure 3. The CMM-RBF architecture, with *N* neurons, has three free parameters: the matrix of synaptic weights $W\in C^{P\times N}$, the matrix of center vectors $\Gamma \in C^{N\times P}$ and the vector of variances $\sigma^{2}\in C^{N\times 1}$. Besides the fact that the CMM-RBF is an extension of the PTRBFNN for multiple outputs, the key difference between both architectures is the linear layer which relates the obtained vector of electric currents with the desired radiation pattern.

The output vector of electric currents is then given by

i[u]=W[u]ϕ[u].

Following the complex-valued radial basis function presented in [7], the *n*th neuron output of the CMM-RBF ($\phi_{n}$), for the *q*th steering vector of Ψ, is

(7) ϕn=exp−‖Re{ψq}−Re{γn}‖22Re{σn2}+𝚥exp−‖Im{ψq}−Im{γn}‖22Im{σn2},

where ${\left\|\cdot \right\|}_{2}$ is the operator which returns the Euclidean norm of its argument and Re{·} and Im{·} are the respective real and imaginary parts of their arguments. Additionally, as shown in Figure 3, the output of the neurons can be represented by the vector $\phi ={\left[\phi_{1}\;\phi_{2}\;\cdots \;\phi_{N}\right]}^{T}\in C^{N\times 1}$. This kernel partitioning into real and imaginary components has an important role in avoiding any phase invariance at the output of the neurons. As the steering vector phase is important to define the electric currents to the desired boresight, a complex RBFNN is not suitable for this application. As addressed in [7], the kernel of the complex RBFNN is not partitioned into real and imaginary parts, which implies that the Euclidean norm eliminates the phase component of the input signal. Consequently, a complex RBFNN is only suitable for phase independent systems.

Thus, by means of the steepest descent algorithm, the update of the CMM-RBF free parameters, to the *q*th steering vector, is given by:

$$w_{p,n}\left[u\right]=w_{p,n}\left[u\right]-\eta_{w}\nabla^{w}J_{q}\left[u\right],$$

(8) $$\gamma_{n}\left[u\right]=\gamma_{n}\left[u\right]-\eta_{\gamma }\nabla^{\gamma }J_{q}\left[u\right],$$

$$\sigma_{n}^{2}\left[u\right]=\sigma_{n}^{2}\left[u\right]-\eta_{\sigma }\nabla^{\sigma }J_{q}\left[u\right],$$

in which $\eta_{w}$, $\eta_{\gamma }$ and $\eta_{\sigma }$ are the adaptive steps of $w_{p,n}$, $\gamma_{n}$ and $\sigma_{n}^{2}$, respectively. Also, $\nabla^{w}$, $\nabla^{\gamma }$ and $\nabla^{\sigma }$ are, respectively, the complex gradient operators of $w_{p,n}$, $\gamma_{n}$ and $\sigma_{n}^{2}$.

The CMM-RBF cost function is the same utilized in the LMS algorithm Equation (4). Applying the complex gradient operators ($\nabla^{w}$, $\nabla^{\gamma }$ and $\nabla^{\sigma }$) to Equation (4) yields:

$$\nabla^{w}J_{q}\left[u\right]=-\epsilon_{q}\left[u\right]\psi_{q,p}^{*}\phi_{n}{\left[u\right]}^{*},$$

(9) ∇γJq[u]=−ξn[u](Re{αn[u]}+Im{αn[u]})+ξn[u]*(Re{αn[u]}−Im{αn[u]})],

∇σJq[u]=−ξn[u](Re{βn[u]}+Im{βn[u]})+ξn[u]*(Re{βn[u]}−Im{βn[u]})],

in which $\epsilon_{q}\left[u\right]=d_{q}-g_{q}\left[u\right]$, as in the LMS algorithm. The synaptic transmittance of the *n*th neuron $\xi_{n}\left[u\right]$ is

(10) $$\xi_{n}\left[u\right]=\epsilon_{q}{\left[u\right]}^{*}\sum_{p=1}^{P}\psi_{q,p}w_{p,n}\left[u\right].$$

The *n*th element of the vector of weighted kernel $\beta \left[u\right]={\left[\beta_{1}\left[u\right]\;\beta_{2}\left[u\right]\;\cdots \;\beta_{N}\left[u\right]\right]}^{T}\in C^{N\times 1}$ is:

(11) βn[u]=Re{ϕn[u]}‖Re{ψq}−Re{γn[u]}‖22Re{σn2[u]}2+𝚥Im{ϕn[u]}‖Im{ψq}−Im{γn[u]}‖22Im{σn2[u]}2.

Similarly, the matrix of weighted centers is represented as $A\left[u\right]={\left[\alpha_{1}^{T}\left[u\right]\;\alpha_{2}^{T}\left[u\right]\;\cdots \;\alpha_{N}^{T}\left[u\right]\right]}^{T}\in C^{N\times P}$, where the *n*th vector of A[u] is

(12) αn[u]=Re{ϕn[u]}(Re{ψq}−Re{γn[u]})Re{σn2[u]}+𝚥Im{ϕn[u]}(Im{ψq}−Im{γn[u]})Im{σn2[u]}.

Finally, applying Equation (9) into Equation (8), the update of the CMM-RBF free parameters for beamforming is expressed as follows:

$$w_{p,n}\left[u\right]=w_{p,n}\left[u\right]+\eta_{w}\epsilon_{q}\left[u\right]\psi_{q,p}^{*}\phi_{n}{\left[u\right]}^{*},$$

(13) γn[u]=γn[u]+ηγ[ξn[u](Re{αn[u]}+Im{αn[u]})+ξn[u]*(Re{αn[u]}−Im{αn[u]})],

σn2[u]=σn2[u]+ησ[ξn[u](Re{βn[u]}+Im{βn[u]})+ξn[u]*(Re{βn[u]}−Im{βn[u]})].

Generalizing Equation (13) to matrix structures, results in:

$$W\left[u\right]=W\left[u\right]+\eta_{w}\epsilon_{q}\left[u\right]\psi_{q}^{*}\phi {\left[u\right]}^{H},$$

(14) Γ[u]=Γ[u]+ηγ[Ξ[u](Re{A[u]}+Im{A[u]})+Ξ[u]H(Re{A[u]}−Im{A[u]})],

σ2[u]=σ2[u]+ησ[Ξ[u](Re{β[u]}+Im{β[u]})+Ξ[u]H(Re{β[u]}−Im{β[u]})].

in which ${\left[\cdot \right]}^{H}$ denotes the transpose conjugate operator and Ξ[u] is the diagonal matrix of synaptic transmittance:

(15) $$\Xi \left[u\right]=\left[\begin{array}{cccc} \xi_{1}\left[u\right] & 0 & \cdots & 0\\ 0 & \xi_{2}\left[u\right] & \cdots & 0\\ \vdots & \vdots & \ddots & \vdots \\ 0 & 0 & \cdots & \xi_{N}\left[u\right] \end{array}\right]\;\in \;C^{N\times N}.$$

Although Equation (14) minimizes the error between the obtained and the desired radiation patterns, as the neurons are dependent on exponential functions, a risk of instability is assumed if the exponential argument is positive. In order to circumvent this issue, based on Theorem A1 (Appendix A), the real and imaginary parts of each scalar component of the vector of variances is lower bounded by the limit μ>0, which, consequently, bounds the real and imaginary parts of the neurons output from 0 to 1.

As in the LMS algorithm, each training epoch is composed of *Q* updates, due to the *Q* sensors, and at the beginning of each training epoch W[u]≜W[u−1], Γ[u]≜Γ[u−1], and $\sigma^{2}\left[u\right]≜\sigma^{2}\left[u-1\right]$. However, for u=0, the CMM-RBF free parameters are initialized following some criterion defined by the user (e.g., based on the probability distribution of the input data).

## 5. Simulations and Discussion

The proposed complex MIMO RBF architecture was evaluated and compared with the LMS algorithm for beamforming at 2.4 GHz. Simulations consider two array arrangements with dipoles of equal length l=0.5λ=6.25 cm and separation distances $s_{d}=0.25\lambda =3.12$ cm: (1) uniform and circular array (UCA) with P=6 dipoles; and (2) 2D-grid array (2D-GA) with P=16 dipoles. For each array arrangement, the matrix of steering vectors is computed via Algorithm A1 (Appendix B). The vectors of angular position $\theta \in R^{Q\times 1}$ and $\omega \in R^{Q\times 1}$ have their number of components (*Q*) defined by the number of sensors, which is selected by the user in a manner to well describe the desired radiation pattern. Besides, each component of the desired radiation pattern d is defined between 0 and 1, in which 0 is used for nulls and 1 is used for the maximum value of the radiation pattern.

As practical systems may suffer severe signal distortion due to occurrence of nonlinearities at the transmitter high power amplifier, based on [32], the nonlinearities are introduced as: $g_{q}\left[u\right]=\psi_{q}^{T}\left(\rho_{1}i\left[u\right]+\rho_{2}i^{2}\left[u\right]+\rho_{3}i^{3}\left[u\right]\right)$, where $\rho ={\left[\rho_{1}\;\rho_{2}\;\rho_{3}\right]}^{T}={\left[0.8\;0.3\;0.2\right]}^{T}$ is the coefficient vector of nonlinearities, $i^{2}\left[u\right]={\left[i_{1}{\left[u\right]}^{2}\;i_{2}{\left[u\right]}^{2}\;\cdots \;i_{P}{\left[u\right]}^{2}\right]}^{T}$ and $i^{3}\left[u\right]={\left[i_{1}{\left[u\right]}^{3}\;i_{2}{\left[u\right]}^{3}\;\cdots \;i_{P}{\left[u\right]}^{3}\right]}^{T}$. Note that, if $\rho_{1}=1.0$ and $\rho_{2}=\rho_{3}=0.0$, it is the linear case, since $g_{q}\left[u\right]=\psi_{q}^{T}i\left[u\right]$.

The LMS and the CMM-RBF were implemented using Algorithms A2 and A3 (Appendix B), respectively. The vector of electric currents is initialized with i[0]≜0+𝚥0, for both beamforming techniques. The free parameters of the CMM-RBF are initialized as: W[0]=0+𝚥0; each center vectors of Γ[0] starts with an unique steering vector of Ψ divided by 10; and $\sigma^{2}\left[0\right]=5+𝚥5$. Also, as discussed in Section 4, μ=0.1 to bound the real and imaginary outputs of the neurons of the CMM-RBF. In order to well represent the set of inputs (steering vectors) into the nonlinear space of the proposed neural network, while maintaining a low computational complexity, we have found by trial and error that N=4 neurons implemented in the CMM-RBF is sufficient. By trial and error, the adaptive steps of the LMS and the CMM-RBF were found to be around $\eta_{l}=0.1$, $\eta_{w}=0.7$, $\eta_{\gamma }=0.5$, and $\eta_{\sigma }=0.5$ for the linear and circular arrays. On the other hand, for the 2D-grid array, we found that the adaptive step of the LMS should be reduced to $\eta_{l}=0.035$. These adaptive steps were chosen to maximize the convergence rate and the HPBW, maintaining the side lobe of the radiation pattern smaller than −20 dB.

The performance of the proposed CMM-RBF is evaluated against LMS by means of the resulting MSE and HPBW for a specified boresight $\hat{\omega }$. The MSE is computed considering an average of 500 simulations for each $\hat{\omega }$. In addition, via the 500 MSE computations, the mean of the normalized radiation patterns were selected to graphically illustrate the HPBW.

### 5.1. Uniform and Circular Array

**Figure 4:**
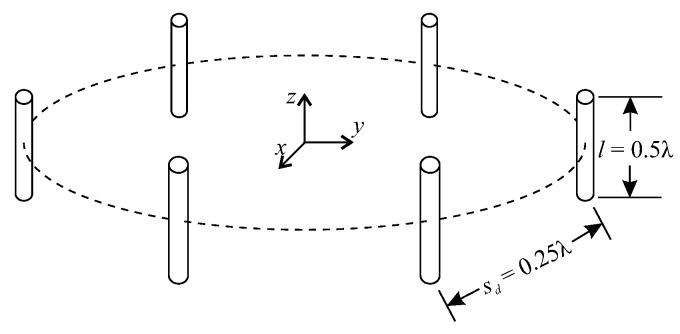
Uniform and circular array (UCA) with six dipoles, where *l* is the dipole length and $s_{d}$ is the distance between dipoles.

In this scheme, six dipoles of equal length are spatially distributed as shown in Figure 4. Both of the presented beamforming techniques share the same Q=10 restrictions, given by the position of the sensors depicted in Table A1 (Appendix C). Restrictions were generalized for the boresight angle $\hat{\omega }$.

**Figure 5:**
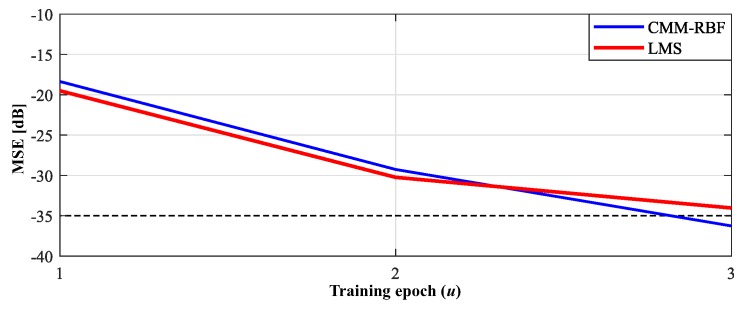
Mean squared error (MSE) of the complex multiple-input multiple-output radial basis function neural network (CMM-RBF) and least mean square (LMS) algorithms for three training epochs: uniform and circular array (UCA) with target $\hat{\omega }=$ 160°.

Figure 5 presents the evolution of the simulated mean squared error (MSE) of the LMS and CMM-RBF algorithms for UCA with $\hat{\omega }=160^{{}^{\circ}}$. As a fast convergence rate characteristic is extremely important for LEO satellites, it is assumed here that convergence for both algorithms is reached when the respective MSE drops below −35 dB. Notice that the MSE for both algorithms decrease similarly up to the second training epoch, after that, the MSE of the CMM-RFB decreases at a much faster rate. It is clear, therefore, that the CMM-RBF achieves a faster convergence rate and delivers a 2.3 dB lower residual MSE (after only three epochs) in comparison with the LMS.

**Figure 6:**
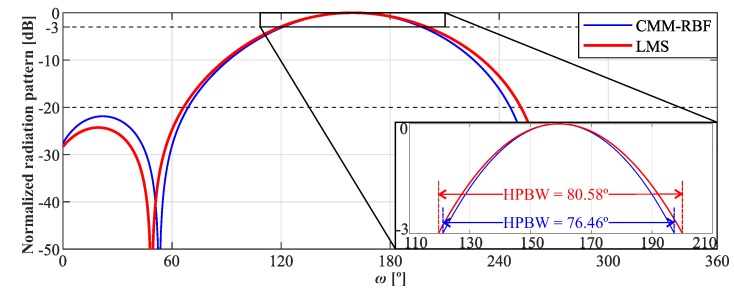
Normalized radiation pattern of the complex multiple-input multiple-output radial basis function neural network (CMM-RBF) and least mean square (LMS) algorithms for the uniform and circular array (UCA) with θ= 90° and $\hat{\omega }=$ 160°.

Figure 6 shows the radiation diagram of the LMS and CMM-RBF algorithms for $\theta =90^{{}^{\circ}}$ and $\hat{\omega }=160^{{}^{\circ}}$. One may note that both algorithms presented similar performance, however, the CMM-RBF enhanced HPBW by 1.12° in comparison with LMS.

In addition, maintaining the same initialization scheme, but varying the boresight angle from $0^{{}^{\circ}}$ to $360^{{}^{\circ}}$, in steps of $10^{{}^{\circ}}$, a number of 500 simulations were performed for each boresight. The mean HPBW and MSE of each boresight is used to compute the statistical results presented in Table 1. The proposed CMM-RBF algorithm is able to enhance the HPBW of the antenna arrays by about 4.15° when operating under the same conditions as the LMS, taking into account the nonlinearities of the transmitter power amplifier.

### 5.2. 2D-Grid Array

**Figure 7:**
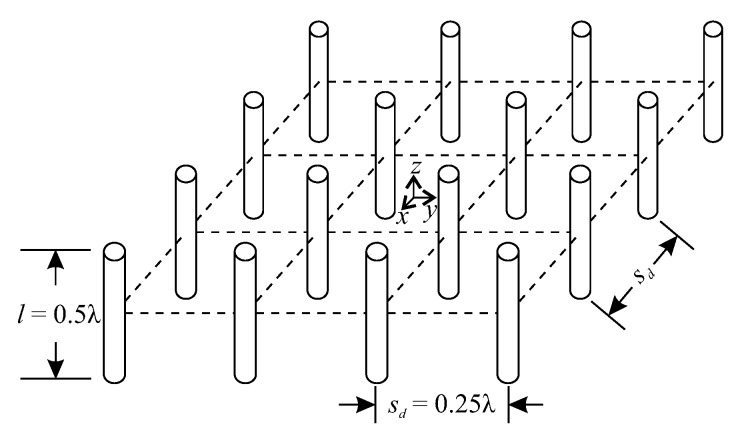
2D-grid array (2D-GA) with 16 dipoles, where *l* is the dipole length and $s_{d}$ is the distance between dipoles.

In this scheme, 16 dipoles of equal length are spatially distributed in a squared grid, as shown in Figure 7. The Q=10 restrictions are depicted in Table A2 (Appendix C).

**Figure 8:**
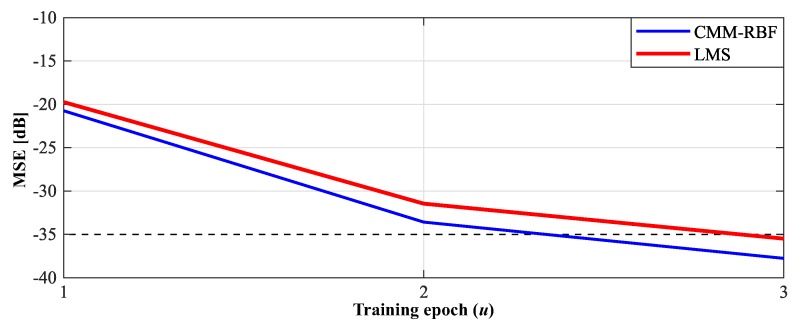
Mean squared error (MSE) of the complex multiple-input multiple-output radial basis function neural network (CMM-RBF) and least mean square (LMS) algorithms for three training epochs: 2D-grid array (2D-GA) with target $\hat{\omega }=$ 160°.

Figure 8 presents the evolution of the simulated mean squared error (MSE) of the LMS and CMM-RBF algorithms for 2D-GA with $\hat{\omega }=160^{{}^{\circ}}$. Convergence is reached when the MSE drops below −35 dB. In this case, diferently from the UCA simulation, the MSE of the CMM-RFB decreases at a faster rate since the first training epoch. At the 3rd epoch, the CMM-RBF delivers a 2.27 dB lower residual MSE in comparison with the LMS.

**Figure 9:**
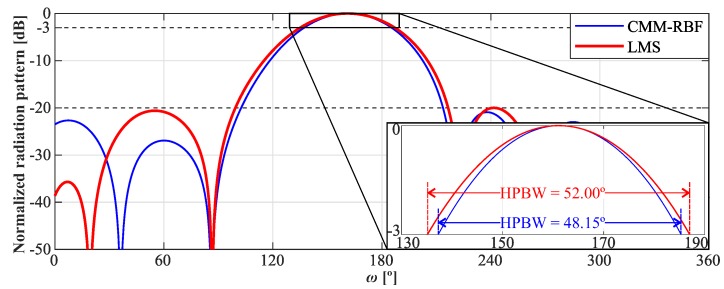
Normalized radiation pattern of the complex multiple-input multiple-output radial basis function neural network (CMM-RBF) and least mean square (LMS) algorithms for the 2D-grid array (2D-GA) with θ= 90° and $\hat{\omega }=$ 160°.

Figure 9 shows the radiation diagram of the LMS and CMM-RBF algorithms for $\theta =90^{{}^{\circ}}$ and $\hat{\omega }=160^{{}^{\circ}}$. Both algorithms presented similar performances, however, the CMM-RBF enhanced HPBW by 3.85° in comparison with LMS.

Moreover, varying the boresight angle from $0^{{}^{\circ}}$ to $360^{{}^{\circ}}$, in steps of $10^{{}^{\circ}}$, a number of 500 simulations were also performed for each boresight and for both architectures, as in Table 1. Results for the mean and standard deviation are presented in Table 2. As in the UCA, the proposed CMM-RBF algorithm is able to enhance the HPBW of the 2D-grid array by about 4.15° when operating under the same conditions as the LMS, taking into account the nonlinearities of the transmitter power amplifier. In 77.78% of the simulations, the CMM-RBF was able to maintain the side lobes of the radiation pattern smaller than −20 dB; on the other hand, the LMS only achieved this condition for 44.45% of the simulations.

## 6. Conclusions

This work presented a novel artificial neural network beamforming scheme for wireless transmission affected by nonlinearities of the transmitter power amplifier. The proposed complex MIMO RBFNN (CMM-RBF) is an extension of the PTRBFNN, used for channel equalization, which is able to handle multiple complex-valued outputs, keeping the phase transmittance information. With the proposed architecture it is possible to simultaneously achieve fast convergence rate, lower MSE and enhanced HPBW in comparison with the LMS in nonlinear scenarios.

The performance of the proposed approach was compared with the LMS for beamforming with a uniform and circular array (6 dipoles of equal length) and a 2D-grid array (16 dipoles of equal length), operating at 2.4 GHz. A set of 36 boresight angles was evaluated and from each generated radiation diagram the respective HPBW was obtained; besides, for each boresight the convergence rate was estimated for the minimum number of epochs possible, in order to obtain a faster tracking.

The proposed MIMO artificial neural network architecture proved to be robust, independent of the boresight angle, achieving faster convergence rate in only three training epochs (after crossover with the LMS) and reducing the MSE by about 2 dB when compared with the LMS algorithm. As for the HPBW, the results obtained with the CMM-RBF are 4.15° better than with the LMS. As the nonlinear behavior of the transmitter power amplifier becomes more prominent when the number of antennas is increased and when operating with a more complex architecture (2D-GA), the LMS beamforming presented a poor performance regarding side lobe restrictions, correctly operating in less than half of the cases. Conversely, the CMM-RBF achieved the side lobe restrictions in more than three quarters of the simulations.

The proposed algorithm finds potential applications in some configurations of the next-generation wireless systems and in satellite communications. For LEO satellites, the CMM-RBF can be implemented using low-power graphical processing units (LPGPUs), taking advantage of the neuron’s parallelism. Thus, in the proposed architecture the CMM-RBF can work with low-power consumption, with the ability to handle the distortions of nonlinear power amplifiers while maintaining a fast convergence rate. It should be emphasized that a fast convergence characteristic is extremely important for LEO satellites, since they orbit non-stationarily at low altitudes.

## Figures and Tables

**Table 1:** Half-power beamwidth (HPBW) and mean squared error (MSE) statistical results for the uniform and circular array (UCA), varying the boresight angle from 0° to 360°.

Algorithm	mean HPBW	std HPBW	mean MSE	std MSE
LMS	81.00°	0.52°	−33.94 dB	0.15 dB
CMM-RBF	76.85°	0.64°	−36.05 dB	0.37 dB

**Table 2:** Half-power beamwidth (HPBW) and mean squared error (MSE) statistical results for the 2D-grid array, varying the boresight angle from 0° to 360°.

Algorithm	Mean HPBW	Std HPBW	Mean MSE	Std MSE
LMS	52.57°	0.88°	−35.36 dB	1.28 dB
CMM-RBF	48.42°	0.33°	−37.80 dB	1.04 dB

## Appendix A

**Theorem** **A1.**
*For any two vectors of same size* $x,y\in R^{n\times 1}$ *and a scalar* z∈R *, a kernel function* $f\left(x,y,z\right)≜\exp\left\{-\frac{{\left\|x-y\right\|}_{2}^{2}}{z}\right\}$ *is bounded between 0 and 1, if* z>0 *.*

**Proof.** By the properties of nonnegativity and definiteness, which state that the norm is always nonnegative (see [33], p. 46), let $d={\left\|x-y\right\|}_{2}\geq 0$. Also, let $g\left(d\right)=-d^{2}/z$, where *z* is a constant scalar. As g(d) is continuous for any z>0 (see [34], p. 43), its natural domain is (−∞,+∞). In order to define the extremes of the image of g(d) it is necessary to assess the extremes of the natural domain and the second derivative test theorem (see [34], p. 186) of g(d). Firstly, we verify the extremes of g(d) for z>0:

$$\lim_{d\to -\infty }g\left(d\right)=-\infty ,$$

$$\lim_{d\to +\infty }g\left(d\right)=-\infty .$$

We then analyze the first derivative, equating its result to zero:

$$\frac{\partial g(d)}{\partial d}=-\frac{2d}{z}=0,$$

which implies that g(d) has only one point of minimum, maximum, or inflection. Due to this, verifying the second derivative of g(d) for d=0:

$$\frac{\partial^{2}g\left(d\right)}{\partial d^{2}}=-\frac{2}{z}<0,$$

thus, d=0 is the maximum point of g(d), which yields g(0)=0 and, consequently, g(d)≤0. Finally, with h(g)≜exp(g(d)), we can verify the image of h(g):

$$\lim_{g(d)\to -\infty }h\left(g\left(d\right)\right)=0,$$

$$\lim_{g(d)\to 0}h\left(g\left(d\right)\right)=1,$$

which yields h(g(d))∈(0,1] ∀d∈R and z>0. □

## Appendix B

**Algorithm A1** Steering vectors computation.
1: **procedure**ComputeSteeringVectors(C, θ, ω, *Q*, *f*, *l*) 2: c←299,792,458 m/s 3: λ←c/f 4: Ω← compute Equation (3) with θ and ω 5: $V\leftarrow \exp(𝚥2\pi \lambda^{-1}\Omega C)$ 6: **for** q←1,Q **do** 7: $\zeta_{q}\leftarrow \sin{\left(\theta_{q}\right)}^{-1}\left[\cos\left(\pi l\cos\left(\theta_{q}\right)\lambda^{-1}\right)-\cos\left(\pi l\lambda^{-1}\right)\right]$ 8: **end for** 9: ζ← compute Equation (1) with $\zeta_{1}\;\zeta_{2}\;\cdots \;\zeta_{Q}$ 10: Ψ←ζV 11: **return** Ψ 12: **end procedure**

**Algorithm A2** LMS for beamforming.
1: **procedure**LMS(i[0], Ψ, d, $\eta_{l}$, ρ, *Q*, *U*) 2: **for** u←1,U **do** 3: i[u]←i[u−1] 4: **for** q←1,Q **do** 5: $i^{2}\left[u\right]\leftarrow {\left[i_{1}{\left[u\right]}^{2}\;i_{2}{\left[u\right]}^{2}\;\cdots \;i_{P}{\left[u\right]}^{2}\right]}^{T}$ 6: $i^{3}\left[u\right]\leftarrow {\left[i_{1}{\left[u\right]}^{3}\;i_{2}{\left[u\right]}^{3}\;\cdots \;i_{P}{\left[u\right]}^{3}\right]}^{T}$ 7: $g_{q}\left[u\right]\leftarrow \psi_{q}^{T}\left(\rho_{1}i\left[u\right]+\rho_{2}i^{2}\left[u\right]+\rho_{3}i^{3}\left[u\right]\right)$ 8: $\epsilon_{q}\left[u\right]\leftarrow d_{q}-g_{q}\left[u\right]$ 9: $i\left[u\right]\leftarrow i\left[u\right]+\eta_{l}\epsilon_{q}\left[u\right]\psi_{q}^{*}$ 10: **end for** 11: **end for** 12: **return** i[U] 13: **end procedure**

**Algorithm A3** CMM-RBF for beamforming.
1: **procedure**CMM-RBF(i[0], W[0], Γ[0], $\sigma^{2}\left[0\right]$, $\eta_{w}$, $\eta_{\gamma }$, $\eta_{\sigma }$, μ, ρ, Ψ, d, *Q*, *U*, *N*) 2: **for** u←1,U **do** 3: i[u], W[u], Γ[u], $\sigma^{2}\left[u\right]\leftarrow i\left[u-1\right]$, W[u−1], Γ[u−1], $\sigma^{2}\left[u-1\right]$, respectively. 4: **for** q←1,Q **do** 5: $i^{2}\left[u\right]\leftarrow {\left[i_{1}{\left[u\right]}^{2}\;i_{2}{\left[u\right]}^{2}\;\cdots \;i_{P}{\left[u\right]}^{2}\right]}^{T}$ 6: $i^{3}\left[u\right]\leftarrow {\left[i_{1}{\left[u\right]}^{3}\;i_{2}{\left[u\right]}^{3}\;\cdots \;i_{P}{\left[u\right]}^{3}\right]}^{T}$ 7: $g_{q}\left[u\right]\leftarrow \psi_{q}^{T}\left(\rho_{1}i\left[u\right]+\rho_{2}i^{2}\left[u\right]+\rho_{3}i^{3}\left[u\right]\right)$ 8: $\epsilon_{q}\left[u\right]\leftarrow d_{q}-g_{q}\left[u\right]$ 9: **for** n←1,N **do** 10: $\phi_{n}\left[u\right]\leftarrow$ compute Equation (7) with $\gamma_{n}\left[u\right]$, $\sigma_{n}^{2}\left[u\right]$ and $\psi_{r}\left[u\right]$ 11: $\xi_{n}\left[u\right]\leftarrow$ compute Equation (10) with $\psi_{q}$, W[u], and $\epsilon_{q}\left[u\right]$ 12: $\beta_{n}\left[u\right]\leftarrow$ compute Equation (11) with $\psi_{q}$, $\gamma_{n}\left[u\right]$, $\phi_{n}\left[u\right]$, and $\sigma_{n}^{2}$ 13: $\alpha_{n}\left[u\right]\leftarrow$ compute Equation (12) with $\psi_{q}$, $\gamma_{n}\left[u\right]$, $\phi_{n}\left[u\right]$, and $\sigma_{n}^{2}$ 14: **end for** 15: Ξ[u]← compute Equation (15) with $\xi_{1}\left[u\right]\;\xi_{2}\left[u\right]\;\cdots \;\xi_{N}\left[u\right]$ 16: $W\left[u\right]\leftarrow W\left[u\right]+\eta_{w}\epsilon_{q}\left[u\right]\psi_{q}^{*}\phi {\left[u\right]}^{H}$ 17: Γ[u]←Γ[u]+ηγ[Ξ[u](Re{A[u]}+Im{A[u]})+Ξ[u]H(Re{A[u]}−Im{A[u]})] 18: σ2[u]←σ2[u]+ησ[Ξ[u](Re{β[u]}+Im{β[u]})+Ξ[u]H(Re{β[u]}−Im{β[u]})] 19: **for** n←1,N **do** 20: **if** Re{σn2[u]}<μ **then** 21: Re{σn2[u]}←μ 22: **end if** 23: **if** Im{σn2[u]}<μ **then** 24: Im{σn2[u]}←μ 25: **end if** 26: **end for** 27: $i\left[u\right]\leftarrow W\left[u\right]\phi_{q}\left[u\right]$ 28: **end for** 29: **end for** 30: **return** i[U] 31: **end procedure**

## Appendix C

**Table A1:** Beamformer restrictions of the uniform and circular array (UCA) with 6 dipoles: $\hat{\omega }$ is the generalized boresight.

Sensor (r)	Zenith $\theta_{r}$	Azimuth $\omega_{r}$	Desired Radiation Pattern $d_{r}$
1	90°	$\hat{\omega }$	1.0
2	90°	$\hat{\omega }+$ 180°	0.0
3	90°	$\hat{\omega }+$ 50°	0.5
4	90°	$\hat{\omega }-$ 50°	0.5
5	90°	$\hat{\omega }+$ 60°	0.2
6	90°	$\hat{\omega }-$ 60°	0.2
7	90°	$\hat{\omega }+$ 115°	0.0
8	90°	$\hat{\omega }-$ 115°	0.0
9	90°	$\hat{\omega }+$ 155°	0.0
10	90°	$\hat{\omega }-$ 155°	0.0

**Table A2:** Beamformer restrictions of the 2D-grid array (2D-GA) with 16 dipoles: $\hat{\omega }$ is the generalized boresight.

Sensor (r)	Zenith $\theta_{r}$	Azimuth $\omega_{r}$	Desired Radiation Pattern $d_{r}$
1	90°	$\hat{\omega }$	1.0
2	90°	$\hat{\omega }+$ 180°	0.0
3	90°	$\hat{\omega }+$ 30°	0.5
4	90°	$\hat{\omega }-$ 30°	0.5
5	90°	$\hat{\omega }+$ 40°	0.2
6	90°	$\hat{\omega }-$ 40°	0.2
7	90°	$\hat{\omega }+$ 70°	0.0
8	90°	$\hat{\omega }-$ 70°	0.0
9	90°	$\hat{\omega }+$ 105°	0.0
10	90°	$\hat{\omega }-$ 105°	0.0
